# New Insights into Human Nondisjunction of Chromosome 21 in Oocytes

**DOI:** 10.1371/journal.pgen.1000033

**Published:** 2008-03-14

**Authors:** Tiffany Renee Oliver, Eleanor Feingold, Kai Yu, Vivian Cheung, Stuart Tinker, Maneesha Yadav-Shah, Nirupama Masse, Stephanie L. Sherman

**Affiliations:** 1 Department of Human Genetics, Emory University School of Medicine, Atlanta, Georgia, United States of America; 2 Department of Human Genetics, Graduate School of Public Health, University of Pittsburgh, Pittsburgh, Pennsylvania, United States of America; 3 Department of Biostatistics, Graduate School of Public Health, University of Pittsburgh, Pittsburgh, Pennsylvania, United States of America; 4 Division of Cancer Epidemiology and Genetics, National Cancer Institute, Bethesda, Maryland, United States of America; 5 Departments of Pediatrics and Genetics, University of Pennsylvania, Philadelphia, Pennsylvania, United States of America; Stowers Institute for Medical Research, United States of America

## Abstract

Nondisjunction of chromosome 21 is the leading cause of Down syndrome. Two risk factors for maternal nondisjunction of chromosome 21 are increased maternal age and altered recombination. In order to provide further insight on mechanisms underlying nondisjunction, we examined the association between these two well established risk factors for chromosome 21 nondisjunction. In our approach, short tandem repeat markers along chromosome 21 were genotyped in DNA collected from individuals with free trisomy 21 and their parents. This information was used to determine the origin of the nondisjunction error and the maternal recombination profile. We analyzed 615 maternal meiosis I and 253 maternal meiosis II cases stratified by maternal age. The examination of meiosis II errors, the first of its type, suggests that the presence of a single exchange within the pericentromeric region of 21q interacts with maternal age-related risk factors. This observation could be explained in two general ways: 1) a pericentromeric exchange initiates or exacerbates the susceptibility to maternal age risk factors or 2) a pericentromeric exchange protects the bivalent against age-related risk factors allowing proper segregation of homologues at meiosis I, but not segregation of sisters at meiosis II. In contrast, analysis of maternal meiosis I errors indicates that a single telomeric exchange imposes the same risk for nondisjunction, irrespective of the age of the oocyte. Our results emphasize the fact that human nondisjunction is a multifactorial trait that must be dissected into its component parts to identify specific associated risk factors.

## Author Summary

Nondisjunction occurs when chromosomes fail to segregate during meiosis; when this happens, gametes with an abnormal number of chromosomes are produced. The clinical significance is high: nondisjunction is the leading cause of pregnancy loss and birth defects. We have studied trisomy 21 using DNA from individuals with Down syndrome and their parents to identify mechanisms underlying nondisjunction. The results from these studies show that altered patterns of recombination, e.g., no exchange, a single telomeric exchange and a single pericentromeric exchange, were associated with nondisjunction of chromosome 21 within the oocyte. In this report, we stratified maternal cases of trisomy 21 by the type of nondisjunction error (meiosis I or meiosis II) and by maternal age (ages <29, 29–34 and >34 years) and examined both the number and location of recombination by age group. Our results suggest that the risk imposed by the absence of exchange or by a single telomeric exchange is the same, irrespective of the age of the oocyte. In contrast, the risk imposed by a single pericentromeric exchange increases with increasing maternal age. These findings, put into the context of proteins involved in the meiotic process, have enabled us to further understand mechanisms underlying nondisjunction.

## Introduction

The overwhelming majority of trisomy 21, or Down syndrome, is caused by the failure of chromosomes to separate properly during meiosis, also known as chromosome nondisjunction. As nondisjunction is the leading cause of pregnancy loss, mental retardation and birth defects, it is imperative that we understand the biology underlying this phenomenon. Characteristics of chromosome 21 nondisjunction are typical of many of the other human autosomes. That is, the overwhelming majority are due to errors during oogenesis: at least 90% of cases of chromosome 21 nondisjunction are due to maternal meiotic errors [1],[2]. In addition, among these maternal errors, the majority occur during meiosis I (MI) [3],[4]. It has been well established that increased maternal age, the most significant risk factor for nondisjunction, is associated specifically with errors occurring during oogenesis. Interestingly, for chromosome 21 nondisjunction, advanced maternal age is associated with both maternal MI and meiosis II (MII) errors [5].

The timing of meiosis in the human female suggests risk factors that may be involved in chromosome nondisjunction. Meiosis is initiated at about 11–12 weeks of gestation and, after pairing, synapsis and recombination, arrests in prophase I until just prior to ovulation. At that time, the oocyte completes MI and progresses to metaphase II where it remains until it is fertilized and the meiotic process is completed. Thus, homologous chromosomes are arrested in prophase I for 10 to 50 years. In contrast, spermatogenesis in the human male begins at puberty and cells entering meiosis move from one stage to the other with no delay. This extended state of arrest in oocyte formation is hypothesized to be associated with the increased prevalence of maternal nondisjunction.

Chiasmata function to stabilize paired homologous chromosomes (tetrads) during MI along with sister chromatid and centromere cohesion. They also help to properly orient homologous chromosomes on the meiotic spindle [5]. A proportion of nondisjunction is associated with failure of homologues to pair or to recombine, leading to an increased risk for homologue malsegregation during MI [6]–[9]. In our previous work [10], it was estimated that 45% of maternal MI cases of trisomy 21 did not have an exchange along chromosome 21. We also found that the location of the exchange was associated with nondisjunction: a single exchange near the telomere of 21q increased the risk of maternal MI nondisjunction and the presence of an exchange near the centromere increased the risk for so called MII nondisjunction. This association of a MI event (i.e., recombination) with a MII error in chromosome segregation led us to suggest that MII nondisjoining errors are initiated during MI. To represent this finding, we will refer to MII errors in quotes.

Most recently, we have explored the relationship between maternal age and recombination to gain further insight into potential mechanisms of abnormal chromosome segregation [11]. We compared the frequency and the location of exchanges along 21q between women (or “oocytes”) of various maternal ages who had an infant with Down syndrome due to a maternal MI error. While there was no significant association between maternal age and the overall frequency of exchange, the placement of meiotic exchange differed significantly by maternal age. In particular, single telomeric recombinant events were present in the highest proportion among the youngest age group (80%), while the proportions in the oldest group of women with nondisjoined chromosomes 21 and in women with normally disjoining meiotic events were almost equal (14% and 10%, respectively). We speculated that for young women then, the most frequent risk factor for MI nondisjunction is the presence of a telomeric exchange. As a woman ages, her meiotic machinery is exposed to an accumulation of age-related insults, becoming less efficient/more error-prone. The susceptible telomeric exchange pattern still increases susceptibility to nondisjunction, but now even homologous chromosomes with optimally placed exchanges are at risk. Over time, the proportion of nondisjunction due to normal exchange configurations increases as age-dependent risk factors exert their influence. As a result, the most prevalent exchange profile of nondisjoined oocytes shifts from susceptible to non-susceptible patterns with increasing age of the oocyte.

As mentioned above, our studies also identified an association between the presence of a meiotic exchange within the pericentromeric region of 21q and “MII” nondisjunction [10], but further studies were not possible due to limited sample size. We have now increased our sample size and, for the first time, have been able to investigate the relationship of exchange patterns stratified by maternal age for maternal “MII” cases of trisomy 21. This increase in sample size has also allowed us to refine our analysis of recombination in maternal MI cases by maternal age. These analyses have provided further insight into the complex pathways leading to nondisjunction among oocytes.

## Results

### Maternal MI Nondisjunction

#### Absence of Recombination

Recombination plays a major role in the meiotic process. The presence of a single meiotic exchange helps to facilitate proper alignment of homologous chromosomes on the meiotic spindle. In the absence of this exchange, homologous chromosomes are at risk for mal-segregation during MI. As a result, we have focused on the absence of recombination as a risk factor for maternal MI nondisjunction. We hypothesized that this risk factor would have the same influence on homologue segregation, irrespective of the age of the oocyte (i.e., maternal age). If true, we would expect to observe the proportion of the MI errors with no recombination to be highest in the youngest age group (i.e., the lower prevalence age group with few maternal age-related factors) and to decrease in the older age groups (i.e., those higher prevalence groups with accumulated maternal age risk factors). We used the Armitage test of trend and did not find a significant linear decrease in the proportion of cases with zero recombinants with increasing maternal age (p = 0.32): the proportion of cases with zero recombinant events was highest among the youngest age group, but did not decrease linearly with age (Table 1). In a post-hoc analysis, we compared the proportion of cases with zero recombinants between age groups in a pairwise fashion. Using a simple 2×2 chi-square test, we found that there was a significantly greater proportion of cases with zero recombinant events among the young versus middle age groups (p = 0.006), but not between the young versus old (p = 0.21) or between middle versus old (p = 0.09) age groups.

**Table 1:** Frequency Distribution of Observed Recombinants and Inferred Exchanges for each Meiotic Outcome Group Stratified by Maternal Age Group.

Meiotic outcome group	Maternal age group	Number of observed events	Frequency of observed number recombinants			Frequency of the number inferred exchanges		
			0	1	≥2	0	1	≥2
**MI**								
	Young (<29 yrs)	175	0.70	0.20	0.10	0.47	0.32	0.21
	Mid (29–34 yrs)	197	0.56	0.35	0.10	0.18	0.64	0.19
	Old (>34 yrs)	243	0.64	0.27	0.09	0.27	0.49	0.24
**MII**								
	Young (<29 yrs)	58	–	0.66	0.34	–	0.22	0.78
	Mid (29–34 yrs)	69	–	0.78	0.22	–	0.51	0.49
	Old (>34 yrs)	126	–	0.81	0.19	–	0.57	0.44
**Euploid**								
	All Ages	152	0.52	0.39	0.09	0.20	0.50	0.30

To obtain a better representation of the pattern of exchange at the four strand stage of meiosis and to be able to compare patterns among meiotic outcome groups (i.e., MI, “MII” and euploid), we performed a tetrad analysis. This method infers exchange patterns from the recombination observed within each meiotic outcome group and within each age group (see Materials and Methods). It was necessary to conduct this analysis because not all exchanges that occur at the four-strand stage of meiosis can be observed. These estimates were then compared between groups using methods that have been previously described [7],[12],[13]. The observed data predicted that 47% of the youngest women had tetrads with zero exchanges (referred to as “E0”) compared to 18% of women in the middle age group and 27% of women in the oldest age group (Table 1). Amongst normally segregating chromosomes 21, 20% of women were estimated to have tetrads with zero exchanges (Table 1). Comparison of the overall inferred frequency distributions of the number of exchanges indicated that the youngest group was statistically different from the middle-age group (p = 0.005), the oldest age group (p = 0.05) and the euploid sample (p = 0.03). Other comparisons were not significantly different.

#### Location of Recombination

Our first aim was to confirm our previous finding that a single telomeric exchange was a significant risk factor for MI nondisjunction among women of all ages [11]. If true, we would expect the proportion of MI errors with a single telomeric exchange to be highest in the young group and decrease with age using the same argument as above. Initially, we examined maternal age as a predictor of the location of recombination (as defined by interval location) using linear regression. Only cases exhibiting a maternal MI error and only one observed recombinant event were included in this analysis (n = 169). Results showed that maternal age was significantly correlated with the location of recombination: as maternal age increased, the average location of recombination shifted from the most telomeric interval (interval 6) of 21q toward the middle of the chromosome (p = .045). Thus, we confirmed the pattern that suggests that a single telomeric recombinant is a risk factor for nondisjunction, irrespective of the age of the oocyte.

Tetrad analysis showed the same pattern as did the observed recombination data, but it was more striking: among tetrads with single exchanges (referred to as “E1”), 41% were inferred to occur in the most telomeric interval (interval 6, the most distal 3.8 Mb of 21q) among the youngest group of women. This contrasted to 16% of errors in the middle age group, 9% in oldest age group and 7% in the euploid sample (Table 2). Comparing the entire spatial distribution of single exchanges, the youngest group was marginally different from the middle group (p = 0.10) and statistically significantly different from the oldest group (p = 0.02) and the euploid sample (p = 0.006).

**Table 2:** Spatial Distribution of Inferred Single Exchanges for Each Meiotic Outcome Group Stratified by Maternal Age Group.

Meiotic outcome group	Maternal age group	Interval location of inferred single exchange (centromere to telomere)						
		1	2	3	4	5	6	Average interval
**MI**								
	Young (<29 yrs)	0.08	0.09	0.00	0.05	0.37	0.41	4.77
	Mid (29–34 yrs)	0.02	0.06	0.08	0.23	0.46	0.16	4.53
	Old (>34 yrs)	0.05	0.08	0.09	0.29	0.40	0.09	4.18
**MII**								
	Young (<29 yrs)	0.00	0.10	0.38	0.27	0.23	0.03	3.75
	Mid (29–34 yrs)	0.35	0.21	0.11	0.16	0.17	0.00	2.59
	Old (>34 yrs)	0.40	0.30	0.14	0.08	0.07	0.02	2.25
**Euploid**								
	All Ages	0.02	0.15	0.21	0.28	0.28	0.07	3.87

### Maternal “MII” Nondisjunction

#### Amount of Recombination

As in our MI analysis, we initiated our analyses by examining the frequency distribution of recombination by maternal age. We used only those cases with at least one observed recombinant. As outlined in the Materials and Methods, MII errors with no observed recombination were assumed to be post-zygotic, mitotic errors and were excluded from these analyses. Using the Armitage test of trend, we found a significant linear relationship between the frequency of multiple recombinants and maternal age group (p = 0.03): the proportion of cases with multiple recombinants significantly decreased with increasing age group. We found the same interesting pattern when we used these observed data to infer the exchange pattern among tetrads in each age group: 78% of the “MII” nondisjoined chromosomes 21 in the youngest group had multiple exchanges compared with only 49% and 44% of those in the middle and oldest group and only 38% in the euploid sample (Table 1). Statistical comparisons of the overall frequency distribution among the youngest age group with the two older age groups and the euploid sample were statistically significant (p = 0.02, p = 0.02 and p = 0.0005, respectively) and marginally significant between the middle and older age groups (p = 0.06).

#### Location of Recombination

Our previous studies have shown that recombination is increased within the most proximal 3.5 Mb (interval 1) of 21q in maternal “MII” cases of nondisjunction [10]. We hypothesized that this event would increase the risk for nondisjunction irrespective of maternal age, similar to that found for the single telomeric exchange. If our hypothesis were correct, we would expect the proportion of “MII” errors with a recombinant event in interval 1 to be greatest among the youngest group of women and decrease in the older groups. In order to test this hypothesis, linear regression was performed on “MII” errors with one observed recombinant event (n = 194) using maternal age as a predictor of the location of recombination. We found that maternal age was negatively correlated with the location of recombination (p = 0.004), the opposite of what we predicted. Thus, with increasing maternal age, the average location of recombination in cases with a single recombinant shifted towards to the centromere. Our tetrad analyses further indicated that the shift is from the medial locations along chromosome 21 in the young group to the centromeric intervals in the older groups. In particular when we focused on cases estimated to have a single pericentromeric exchange, 0% of women belonging to the youngest group of women and 2% of those in the euploid group were estimated to have a single exchange in interval 1; the overall spatial distributions were not different from one another (p = 0.95). In contrast, 35% of women in the middle age group and 40% of women in the oldest age group had single exchanges in interval 1 (Table 2). Statistical comparisons indicated that the older age group's overall pattern of exchange was significantly different from the euploid sample (p = 0.0005). Other comparisons did not show statistically significant differences (middle vs. euploid, p = 0.16; young vs. euploid, p = 0.95; young vs. middle, p = 0.44; young vs. old, p = 0.20).

## Discussion

Among normal disjoining maternal meiotic events, exchanges most often occur in the center of 21q [11]. This observation suggests that the presence of a single medially placed exchange is important for normal segregation of homologous chromosomes 21. This pattern is in striking contrast to the chromosomes 21 that have undergone maternal MI or “MII” nondisjunction, where either no exchange occurs or single exchanges occur at the very ends of 21q [8],[10]. In order to better understand the factors that play a role in these recombination-related disjoined events, we have examined both the number and location of recombination along nondisjoined chromosomes 21 stratified by maternal age. In these analyses, maternal age served as a proxy for the age of the oocyte.

First, among normally disjoining chromosomes 21 in oocytes, there was no obvious association between maternal age and the frequency of exchange or the location of exchange along chromosome 21. We did not expect to observe a maternal age association, as our comparison group, taken from the CEPH families, was relatively small compared to Kong et al. [9], the only study that has noted such an association. In that study, it took over 14,000 maternal meiotic events in order to identify that the frequency of exchanges increased with maternal age: an additional two recombinants genome-wide were estimated over a 25 year age span . Thus, the magnitude of the observed association is not on the same scale as that observed for nondisjoined meiotic events. Irrespective, we still must be cautious with our results and emphasize that the sample sizes of meiotic events, particularly those in the older age groups were small (Table S1) and thus limited our ability to detect maternal age associations with recombination.

**Figure 1:**
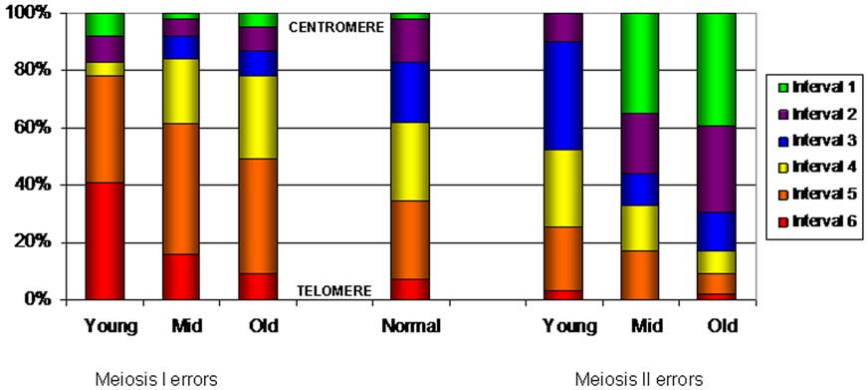
Comparison of Spatial Distributions of Single Exchanges for Meiotic Outcome Groups by Maternal Age. This figure summarizes the data from Table 2. Each color denotes the proportion of single exchanges that are inferred to occur in that specific interval. Proportions were inferred using tetrad analysis and were based on the recombination profiles of meiotic events within age groups and within meiotic outcome group.

**Figure 2:**
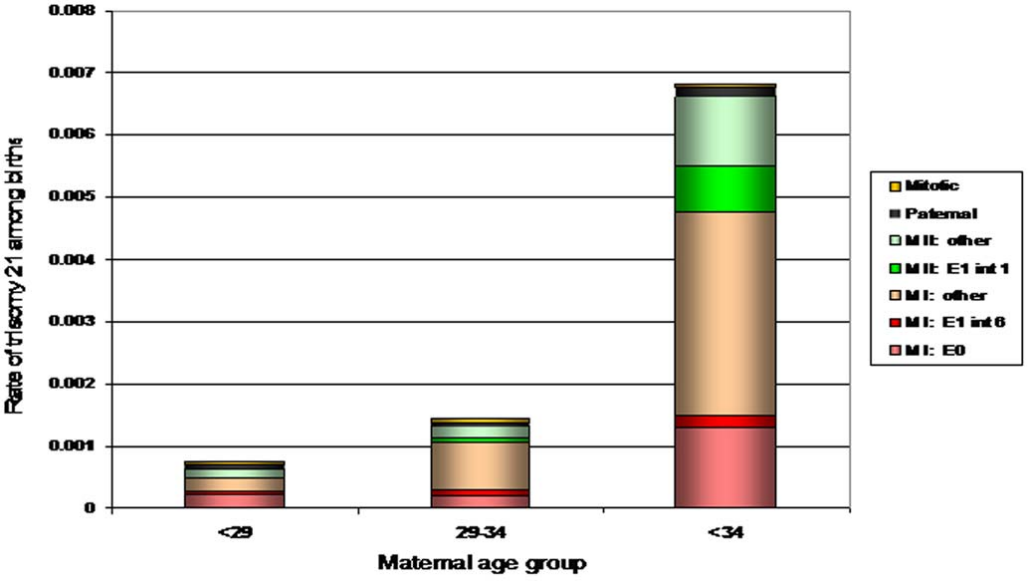
Rate of Trisomy 21 by Maternal age and by Type of Error. Within each maternal age group, the bars indicate the proportion of that rate that is explained by each type of nondisjunction error. See Materials and Methods for the calculation of the proportions.

Whereas there was no obvious maternal age association with recombination patterns among normally disjoining chromosomes 21, there was a significant one among maternal MI and “MII” errors. One set of observations provides evidence for specific recombination patterns being the proximal cause of nondisjunction, while the others suggest an interaction between specific recombination patterns and maternal age-related risk factors. Figure 1 provides an overall summary of our findings related to the spatial distribution of exchanges for MI and “MII” nondisjunction events (using the data from Table 2). In Figure 2, we interpret these findings, as well as those associated with the frequency of exchanges (Table 1) within the context of the overall rate of trisomy 21 among women of the three age groups (see Materials and Methods for calculations). In this figure, the overall rate of trisomy 21 among births by maternal age group is represented by the height of each bar and is estimated from Hecht and Hook [14]. Within each bar, the proportion of those rates that are estimated to have a specific origin and recombination pattern is denoted by color.

Here, we have focused on meiosis occurring in the aging oocyte. Several meiotic proteins that function to promote proper chromosome segregation have been shown to degrade with increasing age [15],[16]. This degradation is assumed to lead to increased frequency of nondisjunction; thus, more maternal-age related risk factors for nondisjunction exist among older women compared to younger women. In the analyses presented here, we have compared the pre-disposing recombination patterns among the oocytes with nondisjoined events by maternal age (Figure 1). Our expectation is that some recombination patterns will lead to susceptibility irrespective of other maternal age factors and these will predominate the youngest age group, or that group with no other risk factors. We found that single telomeric exchanges follow this pattern (Figure 2, “MI: E1 int 6”), as reported previously [11]. This type of error represents less than 8% of each maternal age group. This same risk factor has been established in model organisms as well [17]–[19]. Most likely, susceptibility is related to the minimal amount of the sister chromatid cohesion complex remaining distal to the exchange event [20]. Specifically, when the exchange is too far from the kinetechore, this could prevent the biorientation of homologues on the meiotic spindle [18], [21]–[23]. Alternatively, the integrity of the chiasma may be compromised when a minimal amount of cohesin remains to hold homologues together. Thus, bivalents may act as a pair of functional univalents during MI, as has been observed in human oocytes [24],[25].

The results related to lack of exchange are intriguing, although difficult to interpret at this time. We did find that the proportion of E0s was the highest among the youngest group compared with the other two age groups, indicating a maternal-age independent mechanism. However, the proportions did not decrease linearly with age (Table 1). Conservatively, we can state that E0s lead to susceptibility irrespective of the age of the oocyte. However, the non-significant increase in E0 in the older age group causes us to speculate further. As noted in Figure 2 (“MI: E0”), the lack of a linear decrease by age group suggests that a greater proportion of older oocytes at risk for trisomy 21 will have E0 tetrads compared with the other two age groups. Perhaps these results provide preliminary evidence for a secondary mechanism that is age-dependent. In model systems, there are known mutations that lead to increased nondisjunction of E0s. For example, *Drosophila* with mutations in the gene *nod* (*no distributive disjunction*), show increased nondisjunction of non-exchange chromosomes [26]. This observation was the first to suggest a mechanism that functions to ensure the proper segregation of non-exchange homologues. Studies in yeast also provide evidence for such a mechanism [27]. Interestingly, proteins in humans that may have a similar function to those that play a role in the proper segregation of non-exchange homologues in yeast have been shown to be down regulated with increasing ovarian age [15],[16]. Thus, the age-dependent down-regulation of these essential proteins, or others, may lead to the decreased ability to properly segregate non-exchange chromosomes in aging oocytes. However, this is only speculation at this point. More data are needed to determine significance of our preliminary finding.

Interestingly, the analysis of the normally disjoining meiotic events from the CEPH data indicates a large proportion of E0s, 20%. These data are based on genotyping a high density of chromosome 21-specific SNPs among 152 maternal meiotic events [28]. Other studies have used the CEPH families and have obtained similar frequencies of observed recombinants and estimates of E0 frequencies [29],[30]. These data suggest a higher frequency of E0s compared with other studies that have used techniques that examine tetrads more directly, such as chiasma counts or MLH1 counts. For example, Tease et al. [31] identified three E0 chromosome 21 bivalents out of a total of 86 counted. However, all 86 oocytes analyzed came from only one ovary. As variation in recombination rates among women is well established [28],[32], we need to be careful in drawing conclusions about the difference in estimates of E0 using MLH1 counts versus linkage studies. Nevertheless, future studies are required to determine if the frequency of E0s is significantly different from zero for chromosomes 21 in oocytes (e.g., using MLH1 counts) and in transmissions to births (e.g., linkage studies), each representing a different time point in oocyte development. These studies will complement those among nondisjoined events to determine if a distributive pairing system similar to those in model systems exists in humans.

The other established susceptibility pattern that is associated with an increased risk for “MII” nondisjunction is the presence of a single exchange within the most proximal 5.2 Mb of 21q. When we compared such events among age groups, we observed an enrichment of pericentromeric exchanges in the oldest age group of “MII” nondisjoined chromosomes 21 as summarized in Figure 1. This leads to a greater proportion of trisomy 21 cases among older women being related to pericentromeric exchanges (Figure 2, “MII: E1 int 1”). This pattern can be explained in two different ways: 1) a pericentromeric exchange sets up a suboptimal confirmation that exacerbates the effect of maternal age-related risk factors or 2) a pericentromeric exchange protects the bivalent from maternal-age related risk factors allowing the proper segregation of homologues, but not sister chromatids. An example of the former would be that a pericentromeric exchange compromises proteins involved in centromeric cohesion, exacerbating the normal degradation of this important complex with age. Shugoshin, a protein important in protecting centromere cohesin during MI, would be an obvious target. For example, in yeast cells that were shugoshin deficient, Marston et al. [33]showed that homologous chromosomes segregated to opposite poles in MI, but sister chromatids prematurely separated prior to anaphase II and segregated randomly, sometimes leading to MII nondisjunction . Interestingly, BubR1, the protein required for the localization of shugoshin to the centromere, has been shown to have decreased expression with increasing maternal age in the human female [15],[16]. Perhaps the presence of a pericentromeric exchange exacerbates the degradation of this complex.

Alternatively, a pericentromeric exchange may protect the bivalent from maternal-age related risk factors. The effect of degradation of centromere or sister chromatid cohesin complexes or of spindle proteins with age of the oocyte may lead to premature sister chromatid separation. Perhaps a pericentromeric exchange helps to stabilize the compromised tetrad through MI. This would lead to an enrichment of MII errors among the older oocytes. Although there is no specific model system that points to this mechanism, findings can be interpreted with this mechanism in mind. For example, the effects of a hypomorph of *bubR1* were examined in female meiosis in *Drosophil*a [34]. In mutant females, most chiasmate X chromosome failed to segregate properly at MII, most likely due to premature sister chromatid separation in late MI anaphase or MII. Interestingly, a subtle but repeatable increase in pericentromeric exchanges was identified along such chromosomes.

Lastly, we examined the hypothesis that the number of exchanges may be protective against maternal age-related risk factors. This was first suggested by Robinson et al. [35] , who found that among maternal MI chromosome 15 nondisjunction errors, the age of the mother was significantly increased among cases with multiple recombinants compared with those having zero or only one observed recombinant. From this, the authors suggested that cases with multiple recombinants might be more resistant to nondisjunction because of increased stability of the tetrad over time. Similarly, an analysis of maternal nondisjunction of the X chromosome showed that the mean maternal age of cases with recombination was significantly older than that of cases with no recombination [36]. This same pattern was observed for trisomy 18, although the difference was not statistically significant [37]. For chromosome 21 MI errors, we do not see this pattern. Among the young, middle and older age groups, the observed data infer 40%, 23% and 33% of tetrads have multiple exchanges among our young, middle and old groups respectively (Table 1). Among chromosome 21 “MII” errors, we observe a very different pattern: 78%, 49% and 44% of tetrads have multiple exchanges, respectively. This pattern is opposite of that expected if multiple exchanges were protective. Again, we need to be cautious in our interpretation for the following reason. We have assumed that “MII” cases with no recombination are due to post-zygotic, mitotic events. As shown in Figure 2, these appear to be age-independent events. However, some proportion may be true MII errors with no recombination and we do not have a method to distinguish these alternatives.

We have not discussed our observations related to the placement of multiple recombinants along the nondisjoined chromosomes 21 and the potential effects of altered interference. This is due to the obvious fact that chromosome 21 is small, leading to only a few meiotic events on which we could derive exchange patterns. There were approximately 20 meiotic events in each age category of MI and MII errors. Thus, this type of investigation awaits a larger sample size, or, perhaps, should be based on larger nondisjoined chromosomes (e.g., chromosome 15 or the X chromosome).

The importance of understanding the causes of nondisjunction and the maternal age effect cannot be over-stated. Many women are electing to delay childbearing until their mid-thirties or later, the time at which nondisjunction rates dramatically increase. Irrespective of the exact mechanisms of nondisjunction, our findings indicate that nondisjunction is a complex trait and that there are different risk factors that play a role in age-independent and dependent nondisjunction. The study design for identification of such environmental and genetic risk factors can be guided by our findings. Clearly, examination of nondisjunction events stratified by maternal age, type of error and recombination pattern should increase the power to identify important factors that play a role in chromosome mal-segregation.

## Materials and Methods

### Trisomic Samples

Families with an infant with full trisomy 21 were recruited through a multisite study of risk factors associated with chromosome nondisjunction [2],[8],[10]. Parents and the infant donated a biological sample (either blood or buccal) from which DNA was extracted. All recruitment sites obtained the necessary Institutional Review Board approvals from their institutions.

**Figure 3:**
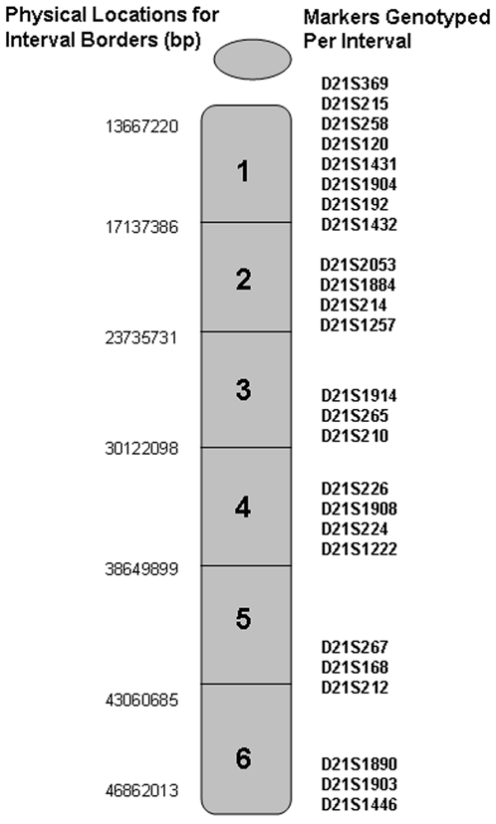
Markers used to Define the Origin of the Meiotic Error and Determine the Recombination Profile. Approximately 21 markers were genotyped on each individual in the study. This information was used first to determine the origin (maternal or paternal and meiosis I or II) of the nondisjoining error. Only cases in which the error was maternal in origin were included in this study. Once the origin of the error was defined, this genotyping information was used to determine the number and location of recombination (i.e., recombination profile). 21q was divided into six intervals of approximately equal physical length. Each observed recombinant was defined as being located in one of six defined intervals.

Only families in which DNA was available from both parents and the child with trisomy 21 were included in the present analysis. A subset of families in the current analysis with maternal MI errors were also included in a previous study [6]. Samples were genotyped for a minimum of 21 short tandem repeat (STR) markers specific to the long arm of chromosome 21 (Figure 3). The most centromeric STR was D21S369 and the most telomeric was D21S1446.

#### Determining the Type of Nondisjoining Error

The parental origin of the nondisjoining error was determined by establishing the contribution of parental alleles to the child with trisomy 21. Only cases of maternal origin were included in our analysis. Once the maternal origin of nondisjunction was established, a core set of markers located in the pericentromic region (D21S369- D21S192, Figure 3) of 21q was used to infer the stage of nondisjunction, MI or “MII”. Specifically, if parental heterozygosity was retained in the trisomic offspring (“nonreduced”), we concluded an MI error. If parental heterozygosity was “reduced” to homozygosity, we concluded an “MII” error. In this assay, we could not distinguish between a conventional MII error, in which sister chromatids fail to separate during anaphase of MII, from an error initiated in MI that is not resolved properly in MII. For example, if sister chromatids prematurely separate in MI, some configurations will lead to both sister chromatids segregating to the same pole in MII. Similarly, if homologues pairs fail to separate in MI and then go through a reductional division at MII, sister chromatids will be present in the resulting oocyte.

When all informative markers in the parent of origin were reduced to homozygosity, the origin of nondisjunction was inferred to be a post-zygotic, mitotic error. In principle, such cases could also be “MII” errors with no recombination. We do not have a method to accurately distinguish these types of errors. We expect that there should be equal numbers of “maternal” and “paternal” errors among such cases and in fact we observed about twice as many “maternal” cases (data not shown). Second, we expect these cases to be maternal age independent and, in fact, find this to be true: the mean maternal age of so-called mitotic cases does not differ from controls [2]. In our current data set, there were 21, 16 and 3 mitotic cases in the young, middle and older age group. To be conservative, we excluded these inferred mitotic cases from our analysis.

### Characterizing the Recombination Profile

Our analysis of the number and location of recombination was restricted to 21q. The long arm of chromosome 21 was divided into six relatively equal physical intervals with interval 1 comprising the most centromeric region of 21q and interval 6 comprising the most telomeric region (Figure 3). The presence of a recombinant event was identified by changes in the status of adjacent informative markers from “reduced” to “nonreduced” (or vice versa). In most cases, the location of recombination was scored as belonging to one of six distinct intervals along 21q. When one of the six intervals was uninformative, but markers defining the two flanking intervals were informative, we included the family. Those with two or more adjacent uninformative intervals were excluded from our analysis. In some instances, the recombinant event could not be located to one specific interval, but instead to one of two adjacent intervals (e.g., interval 1 or interval 2). The location of such events was treated as occurring at the midpoint of the two intervals (e.g., represented as interval 1.5) in most of our analyses (see Statistical Analysis below). Our final analysis included a total of 615 maternal MI cases and 253 maternal “MII” cases of trisomy 21.

#### Euploid Samples

We used the 23 CEPH Utah families that were previously genotyped using 133 SNPs located on the long arm of chromosome 21 [28]. The most centromeric SNP was located at 15,009,674 bp (rs990141) and the most telomeric SNP located at 46,902,239 bp (rs2839337). It is important to point out that the marker set used to genotype these 23 CEPH families was different from that used to genotype the trisomic cases. In addition, offspring within families of the CEPH panel were related, whereas the trisomic cases were not. Thus, there is a slight lack of comparability in the analyses between our euploid and trisomic data, but the differences are minor in comparison to the large differences in recombination observed between the euploid and trisomic samples.

### Characterizing the Recombination Profile

In order to determine the location of recombination along 21q in women who exhibited normal segregation of chromosome 21, the transmission of maternal grandparental SNP genotypes to the maternal offspring was analyzed. A maternal recombinant event was noted when the sharing of SNPs identical by descent switched from one maternal grandparent to the other. Our final analysis included 152 informative maternal meioses.

#### Statistical Analysis

We had two basic traits to characterize with respect to recombination in each dataset: 1) the amount of recombination and 2) the location of recombination. To determine if these characteristics differed among maternal age groups, we used standard statistical methods such as the chi square test of independence, Armitage test of trend and linear regression.

We analyzed the observed recombination data from the euploid, MI and “MII” samples separately since the ability to detect a recombinant event differs between the each of these groups. Within each group, we stratified the samples by the age of the mother at the time of conception, henceforth referred to as maternal age. The three maternal age groups were previously defined [6] and were based on obtaining approximately equal sample sizes in each age group: <29, 29–34 and >34 years of age. This definition applied to the new trisomic data sets did not lead to equal sample sizes due to the shift in maternal age over time. That is, we had more women in the oldest age group (Table 1). Nevertheless, we decided to use this same definition for comparison purposes. For the euploid dataset, this definition led to too few meiotic events in the older age groups. There were 83, 33, and 31 meiotic events for the young, middle, and eldest age groups, respectively. Based on our analyses, we could not detect any statistically significant differences in the amount or location of recombination among maternal age groups in the euploid set. However, our sample sizes limited our ability to do so (see Table S1). Thus, we collapsed the euploid maternal age groups.

As discussed above, direct analyses of observed recombination do not allow for comparisons between the meiotic outcome groups (MI, “MII” and euploid), because exchanges at the four-strand stage have a different probability of being observed as recombination depending on the meiotic outcome. Thus in order to compare meiotic outcome groups, we used the observed recombination data to estimate the number and pattern of exchanges at the four-strand stage of meiosis. We refer to this in the text of the paper as our “tetrad analyses”. These methods have been previously described in detail [7],[12],[13]. Briefly, tetrad exchange pattern frequencies are estimated from observed recombination data using maximum likelihood. Hypothesis tests comparing groups (e.g. MI old vs. euploid) can then be performed using likelihood ratio tests, with the test statistic distributions estimated by bootstrap methods. These methods not only allowed the comparison of meiotic outcome groups, but they also allowed direct comparison of frequencies of single exchanges (or double exchanges) among groups. This allowed us to ask, for example, if single telomeric exchanges are a risk factor (In the observed recombination data these would be confounded with double-exchanges that include a telomeric exchange). For the purposes of these methods we scored ambiguous recombination events as occurring half in each interval (e.g. an exchange that occurred in either interval 1 or interval 2 was scored as 1/2 an exchange in interval 1 and 1/2 an exchange in interval 2).

#### Estimation of the Rate of Nondisjunction Events by Meiotic Error and Exchange Group

To help interpret the results of the exchange patterns observed among the nondisjoined meiotic events, we estimated the rate of each type of nondisjunction error among women in each age group. The overall rate of trisomy 21 among births was estimated to be 1/1320, 1/699, and 1/147 for the three age groups, respectively, using the one-year observed rate estimates from Hecht and Hook [14]. For these approximate estimates, we assumed that all trisomy 21 was due to either meiotic or mitotic errors; that is, we did not include the more rare causes due to translocations and mosaicism. The proportions of the meiotic and mitotic errors types were taken from data collected through the Atlanta Down Syndrome Project [4] and unpublished results. Within each age group, we partitioned that rate of trisomy 21 by meiotic error and then by exchange pattern using estimates from Tables 1 and 2. For example, the rate of MI nondisjunction with a single telomeric exchange was estimated to (1/1320) * 0.63 * 0.32 * 0.41 = 0.000062 for the young age group. This subgroup, thus, explains about 8% of the rate of trisomy 21 in that age group.

## Supporting Information

Table S1 Frequency Distribution of Observed Recombinants and Inferred Exchanges for Euploid Samples. Due to the small sample of normally disjoining meiotic events (n = 152) and the maternal age distribution among those samples, there were a limited number of data points in the oldest two age groups ( Table S1). Although formal statistical tests did not detect any association between maternal age and recombination, the power to detect such an association was low. For these reasons, we collapsed age groups and compared the entire sample to those of the nondisjoining meiotic events. However, to be complete, we have provided the frequency distribution of the number of recombinants below.
(0.03 MB DOC)

## References

[1] Antonarakis SE (1991). Parental origin of the extra chromosome in trisomy 21 as indicated by analysis of DNA polymorphisms. Down Syndrome Collaborative Group.. N Engl J Med.

[2] Freeman SB, Allen EG, Oxford-Wright CL, Tinker SW, Druschel C (2007). The National Down Syndrome Project: design and implementation.. Public Health Rep.

[3] Antonarakis SE, Petersen MB, McInnis MG, Adelsberger PA, Schinzel AA (1992). The meiotic stage of nondisjunction in trisomy 21: determination by using DNA polymorphisms.. Am J Hum Genet.

[4] Yoon PW, Freeman SB, Sherman SL, Taft LF, Gu Y (1996). Advanced maternal age and the risk of Down syndrome characterized by the meiotic stage of chromosomal error: a population-based study.. Am J Hum Genet.

[5] Carpenter AT (1994). Chiasma function.. Cell.

[6] Lamb NE, Sherman SL, Hassold TJ (2005). Effect of meiotic recombination on the production of aneuploid gametes in humans.. Cytogenet Genome Res.

[7] Lamb NE, Feingold E, Sherman SL (1997). Estimating meiotic exchange patterns from recombination data: an application to humans.. Genetics.

[8] Lamb NE, Freeman SB, Savage-Austin A, Pettay D, Taft L (1996). Susceptible chiasmate configurations of chromosome 21 predispose to non-disjunction in both maternal meiosis I and meiosis II.. Nat Genet.

[9] Kong A, Barnard J, Gudbjartsson DF, Thorleifsson G, Jonsdottir G (2004). Recombination rate and reproductive success in humans.. Nat Genet.

[10] Lamb NE, Feingold E, Savage A, Avramopoulos D, Freeman S (1997). Characterization of susceptible chiasma configurations that increase the risk for maternal nondisjunction of chromosome 21.. Hum Mol Genet.

[11] Lamb NE, Yu K, Shaffer J, Feingold E, Sherman SL (2005). Association between maternal age and meiotic recombination for trisomy 21.. Am J Hum Genet.

[12] Yu K, Feingold E (2001). Estimating the frequency distribution of crossovers during meiosis from recombination data.. Biometrics.

[13] Yu K, Feingold E (2002). Methods for analyzing the spatial distribution of chiasmata during meiosis based on recombination data.. Biometrics.

[14] Hecht CA, Hook EB (1996). Rates of Down syndrome at livebirth by one-year maternal age intervals in studies with apparent close to complete ascertainment in populations of European origin: a proposed revised rate schedule for use in genetic and prenatal screening.. Am J Med Genet.

[15] Baker DJ, Jeganathan KB, Cameron JD, Thompson M, Juneja S (2004). BubR1 insufficiency causes early onset of aging-associated phenotypes and infertility in mice.. Nat Genet.

[16] Steuerwald N, Cohen J, Herrera RJ, Sandalinas M, Brenner CA (2001). Association between spindle assembly checkpoint expression and maternal age in human oocytes.. Mol Hum Reprod.

[17] Koehler KE, Boulton CL, Collins HE, French RL, Herman KC (1996). Spontaneous X chromosome MI and MII nondisjunction events in Drosophila melanogaster oocytes have different recombinational histories.. Nat Genet.

[18] Ross LO, Maxfield R, Dawson D (1996). Exchanges are not equally able to enhance meiotic chromosome segregation in yeast.. Proc Natl Acad Sci U S A.

[19] Zetka MC, Rose AM (1995). Mutant rec-1 eliminates the meiotic pattern of crossing over in Caenorhabditis elegans.. Genetics.

[20] Orr-Weaver T (1996). Meiotic nondisjunction does the two-step.. Nat Genet.

[21] Hawley RS, Frazier JA, Rasooly R (1994). Separation anxiety: the etiology of nondisjunction in flies and people.. Hum Mol Genet.

[22] Koehler KE, Hawley RS, Sherman S, Hassold T (1996). Recombination and nondisjunction in humans and flies.. Hum Mol Genet.

[23] Nicklas RB (1974). Chromosome segregation mechanisms.. Genetics.

[24] Angell RR (1995). Meiosis I in human oocytes.. Cytogenet Cell Genet.

[25] Angell RR, Xian J, Keith J, Ledger W, Baird DT (1994). First meiotic division abnormalities in human oocytes: mechanism of trisomy formation.. Cytogenet Cell Genet.

[26] Knowles BA, Hawley RS (1991). Genetic analysis of microtubule motor proteins in Drosophila: a mutation at the ncd locus is a dominant enhancer of nod.. Proc Natl Acad Sci U S A.

[27] Cheslock PS, Kemp BJ, Boumil RM, Dawson DS (2005). The roles of MAD1, MAD2 and MAD3 in meiotic progression and the segregation of nonexchange chromosomes.. Nat Genet.

[28] Cheung VG, Burdick JT, Hirschmann D, Morley M (2007). Polymorphic variation in human meiotic recombination.. Am J Hum Genet.

[29] Bugge M, Collins A, Petersen MB, Fisher J, Brandt C (1998). Non-disjunction of chromosome 18.. Hum Mol Genet.

[30] Lynn A, Kashuk C, Petersen MB, Bailey JA, Cox DR (2000). Patterns of meiotic recombination on the long arm of human chromosome 21.. Genome Res.

[31] Tease C, Hartshorne GM, Hulten MA (2002). Patterns of meiotic recombination in human fetal oocytes.. Am J Hum Genet.

[32] Kong A, Gudbjartsson DF, Sainz J, Jonsdottir GM, Gudjonsson SA (2002). A high-resolution recombination map of the human genome.. Nat Genet.

[33] Marston AL, Tham WH, Shah H, Amon A (2004). A genome-wide screen identifies genes required for centromeric cohesion.. Science.

[34] Malmanche N, Owen S, Gegick S, Steffensen S, Tomkiel JE (2007). Drosophila BubR1 Is Essential for Meiotic Sister-Chromatid Cohesion and Maintenance of Synaptonemal Complex.. Curr Biol.

[35] Robinson WP, Kuchinka BD, Bernasconi F, Petersen MB, Schulze A (1998). Maternal meiosis I non-disjunction of chromosome 15: dependence of the maternal age effect on level of recombination.. Hum Mol Genet.

[36] Thomas NS, Ennis S, Sharp AJ, Durkie M, Hassold TJ (2001). Maternal sex chromosome non-disjunction: evidence for X chromosome-specific risk factors.. Hum Mol Genet.

[37] Fisher JM, Harvey JF, Morton NE, Jacobs PA (1995). Trisomy 18: studies of the parent and cell division of origin and the effect of aberrant recombination on nondisjunction.. Am J Hum Genet.

