# Supplementary material for: New Insights into Human Nondisjunction of Chromosome 21 in Oocytes
Source: PLoS Genet. 2008 Mar 14;4(3):e1000033. doi: 10.1371/journal.pgen.1000033 (PMC2265487; doi:10.1371/journal.pgen.1000033)
Supplement: Table S1 — Frequency Distribution of Observed Recombinants and Inferred Exchanges for Euploid Samples. Due to the small sample of normally disjoining meiotic events (n = 152) and the maternal age distribution among those samples, there were a limited number of data points in the oldest two age groups ( Table S1). Although formal statistical tests did not detect any association between maternal age and recombination, the power to detect such an association was low. For these reasons, we collapsed age groups and compared the entire sample to those of the nondisjoining meiotic events. However, to be complete, we have provided the frequency distribution of the number of recombinants below. (0.03 MB DOC) [file pgen.1000033.s001.doc]

**APPENDIX I**

Due to the small sample of normally disjoining meiotic events (n=152) and the maternal age distribution among those samples, there were a limited number of data points in the oldest two age groups (Table S1). Although formal statistical tests did not detect any association between maternal age and recombination, the power to detect such an association was low. For these reasons, we collapsed age groups and compared the entire sample to those of the nondisjoining meiotic events. However, to be complete, we have provided the frequency distribution of the number of recombinants below.

| **Maternal age group** | **Number of meiotic events** | **Number of observed recombinants** | | | **Number of inferred exchanges** | | |
| --- | --- | --- | --- | --- | --- | --- | --- |
|  |  | **0** | **1** | **≥2** | **0** | **1** | **≥2** |
| Young (<29 yrs) | 83 | 0.54 | 0.36 | 0.1 | 0.26 | 0.4 | 0.34 |
| Mid (29-34 yrs) | 38 | 0.42 | 0.47 | 0.11 | 0.04 | 0.6 | 0.36 |
| Old (>34 yrs) | 31 | 0.58 | 0.39 | 0.03 | 0.22 | 0.65 | 0.13 |
| All Ages Combined | 152 | 0.52 | 0.39 | 0.09 | 0.2 | 0.5 | 0.3 |

Table S1. Frequency Distribution of Observed Recombinants and Inferred Exchanges for Euploid Samples.
